# A Comparative Analysis of the Protection of the Rights of Childhood Cancer Survivors to Education Under Special Education Law

**DOI:** 10.5334/cie.137

**Published:** 2025-04-30

**Authors:** Margaret Flood, Lisa Carey

**Affiliations:** 1 Education Department, Maynooth University, IE; 2 Kennedy Kreiger, Johns Hopkins University, US

**Keywords:** Childhood cancer, Education protections, Inclusion, Equity

## Abstract

Access to equitable education for children treated for cancer is of growing international concern across education, medicine, and related fields. Neurocognitive late effects of childhood cancer and treatment are well established. This impact on cognition results in difficulties with thinking, learning, peer-relationships, and quality of life. Formalized In-School Supports (ISS) can ameliorate the negative impacts of neurocognitive late effects, yet the literature suggests that children treated for cancer often have difficulty accessing these services. This paper reviews the ISS legislation of eight countries regarding protections offered to children treated for cancer and evidence of access to ISS within the literature. The purpose of this review was to look for common barriers for children treated with cancer accessing educational support through ISS. This review identifies gaps between ISS student-focused disability legislation, and practice to inform positive policy change.

Within the past two decades, rates of survivorship of childhood cancer increased to approximately 80% in high-income countries due to access to treatment (WHO, 2021). While rates of survivorship have improved, a growing number of children treated for cancer experience life-long neurocognitive impacts of cancer and treatment (often referred to as neurocognitive late effects) which impact thinking, learning, and quality of life (Erdmann et al., 2021).

Education plays a critical role in the development and well-being of children, but cancer and cancer treatment negatively affect academic and social outcomes (Capurso & Dennis, 2017; Devine et al., 2022; HSE, 2023; Martinez-Santos et al., 2021; Thornton et al., 2022). Research exploring the key factors influencing the education and development of children treated for cancer highlights the challenges they face, and the support systems needed for their educational success. Martinez-Santos et al.’s (2021) systematic literature review showed that, compared to their siblings and peers, children treated for cancer were at risk of lower educational attainment, experienced delays in completing their education and repeating a school year with fewer entering or completing a university or college education. This is often due to the increased rates of neurocognitive dysfunction in childhood cancer survivors which affects attention, memory and executive functions (Adams et al., 2020; HSE, 2023; Thornton et al., 2022). Additionally, long periods of school absences and erratic attendance (Capurso & Dennis, 2017; HSE, 2023) impact opportunities to learn and socialize, highlighting the need to address the logistical demands of cancer treatment such as time spent in hospital, clinics, and recuperating from the side effects of treatment and their impacts on education and schooling (Thornton et al., 2022).While loss of learning and lowered educational outcomes are a key factor, there are also concerns for the social-emotional wellbeing of children treated for cancer that will need to be considered in school. Poor attendance, changes in physical appearance, loss of socializing with peers in addition to loss in learning can significantly impact identity, peer -relations, sense of belonging, and self-esteem, leading to feelings of feeling different to their friends and peers, distress, anxiety and depression (Capurso & Dennis, 2017; Devine et al., 2022; HSE, 2023).

The literature reviewed highlights the multifaceted schooling challenges faced by survivors of childhood cancer. Educators play a vital role in reducing barriers to educational participation and attainment for children treated for cancer. However, there appears to be an uneven application of standardized education supports for children treated with cancer, which poses a barrier to educational success. Receiving formalized In-School Supports (ISS) such as Individualized Education Programs/Plans (IEPs) or Individualized Learning Plans (ILPs) has been shown to reduce barriers to academic and social progress for children treated for cancer (Jacobson et al., 2020). These additional supports often fall under the remit of legislation labeled “special education”, “accommodations for students with disabilities”, “inclusive education,” or “additional education needs” depending on the jurisdiction (see Table 1). In all cases, these are pieces of legislation targeting protected rights and supports for students with recognized disabilities impacting their learning. Special education, disability and equality legislation may address the need for protection of the rights of children treated for cancer to a meaningful education and ensuring access to necessary support services.

**Table 1:** National In-School Supports and Related Disability Legislation.


COUNTRY	TERMINOLOGY	UNDERSTANDING OF/DEFINITION

Australia	Students with Disability	The Disability Standards aim to eliminate, as far as possible, discrimination against persons on the ground of disability in the area of education and training; Schools make reasonable accommodations for students with disabilities. (Australian Department of Education, 2005)

Canada	Depending on the jurisdiction the following terms are used: Special Education Needs Students with disability Diverse learning needs	Each jurisdiction has its own SEN/Inclusion definition and policy based on Article 15 of the Canadian Charter of Rights and Freedoms

Republic of Ireland	Special Education Needs	A continuum of education provision for students with special education needs, ranging from fully inclusive mainstream classes, special classes in mainstream schools to placement in a special school and home tuition in exceptional cases (NCSE, 2024)

United Kingdom: England	Special Education Needs	Special education needs support that can include a special learning programme, additional help from a teacher or assistant, learning in smaller groups, support taking part in activities and communicating with peers, and support with physical or personal care needs. (UK Government, no date)

United Kingdom: Scotland	Special Education Needs/Additional Educational Needs	Additional support for learning grounded in the belief that inclusive education is a fundamental human right, acknowledging diversity, and aiming to ensure that all children and young people can reach their highest potential (Scottish Government, 2017)

United Kingdom: Wales	Special Education Needs/Additional Learning Needs	Special education is provided in various forms, including offering additional support to a child within a regular classroom, educating the child separately in a specially equipped unit within a mainstream school, or in a dedicated special school. (Government of Wales, 2004)

United Kingdom: Northern Ireland	Special Education Needs	Special education provision ensures that students with SEN but without a statement (IEP) are educated in mainstream schools. It also ensures that those with a statement (IEP) are educated in mainstream schools unless this is not the parents’ choice or is insufficient. (Northern Irealnd Assembly, 2016)

United States	Students with Disabilities	Students with disabilities are provided with free, appropriate, public education that includes specialized instruction in the least restricted environment. Their specialized instruction and access to related services is documented in an Individualized Education Program (IEP). (US Department of Education, 2004)


In this paper, we examine the legislation that has an impact of ISS, and requisite measures of five countries to survey the educational protections of children treated for cancer to address the following research questions:

1. What examples of current legislation offers educational rights protections for children treated for cancer?
2. What is the potential application of legal protections to children treated for cancer for each piece of legislation identified?
3. What evidence is there to demonstrate the effectiveness of these laws in providing formalized in-school supports to children treated for cancer?

In addressing these questions, we identify connections and gaps between ISS and related disability legislation and educational supports for children treated for cancer. For each country we summarize ISS and related disability legislation, examine the feasibility in application of these laws to children treated for cancer, and review evidence of effectiveness of ISS access for children treated for cancer based on the available literature.

Given that countries use different terminology and have nuanced differences in approaches to specialized educational supports and related disability rights (Table 1), this paper uses the terminology of ISS policy and provision to mean an inclusive approach to education for children with ISS needs and disabilities. We define ISS as the additional in-school learning and teaching resources required and provided to support inclusive and meaningful access, participation and achievement in their education for children with established needs. Additionally, to streamline the different laws considered within this paper, we refer to special education, disability and equality legislation that impacts the provision of ISS as ISS legislation in general. When discussing country’s individual laws, we use the language of that country.

## A Framework for Comparison

The framework created to compare the legal documents and policies across the specified countries involves a systematic approach encompassing several key dimensions. Firstly, we established a baseline comparison of childhood cancer survival rates, recognizing variations across countries. This serves as a fundamental metric to gauge healthcare outcomes. Secondly, we examined the language(s) of legislation and policy, recognizing the linguistic diversity among the countries under study. Thirdly, we explored the special education, disability rights and equality legislation that impacts ISS provision (ISS legislation) of each country, recognizing the significance of legal frameworks needed to ensure equitable access to education and safeguarding the rights of children with disabilities, in this instance children treated for cancer. This involved identifying key legislative acts and regulations aimed at promoting inclusion and addressing the unique needs of children with disabilities within each country before exploring if and how this legislation extends to children treated with cancer and the actions taken to protect their rights to education. Lastly, we consider the ratification status of the UN Convention on the Rights of Persons with Disabilities, which serves as an international benchmark for promoting and protecting the rights of individuals with disabilities. Understanding the ratification status provides insights into each country’s commitment to upholding international standards and promoting the rights and well-being of individuals with disabilities. By systematically analysing these dimensions, the framework enables a comprehensive comparative analysis of the ISS legal frameworks and policies in the context of children treated with cancer (see Table 2).

**Table 2:** International ISS Laws and Effectiveness for Children Treated for Cancer.


COUNTRY	SURVIVAL RATE*	ISS/DISABILITY RIGHTS LEGISLATION	POTENTIAL APPLICATION TO CHILDREN TREATED FOR CANCER	EFFECTIVENESS	RATIFICATION OF THE UN CRPD**

Australia	83.8%	Disability Discrimination Act (1992), Disability Standards for Education (2005)	Two potential qualifying education disability categories: **a)** “The presence in the body of organisms causing disease or illness” or **b)** “A disorder or malfunction that results in the person learning differently from a person without the disorder or malfunction”	Limited evidence that Australian students with a history of cancer are being granted access to IEPs/ILPs.	17 July 2008

Canada	82%	Canadian Charter of Rights and Freedoms (1981)	Potential qualifying special education disability category: **a)** Communication, Learning Disability: “a neurodevelopmental disorder that persistently and significantly has an impact on the ability to learn and use academic and other skills …difficulties in one or more cognitive processes, such as phonological processing; memory and attention; processing speed; perceptual-motor processing; visual-spatial. ” processing; executive functions (e.g., self-regulation of behavior and emotions, planning, organizing of thoughts and activities, prioritizing, decision making)”	Limited empirical evidence of Canadian students with a history of cancer being granted access to special education and related services.	11 March 2010

Republic of Ireland	81%	Education for Persons with Special Education Needs Act (2004)	No specific special education eligibility criteria fits children treated for cancer, but Students with Educational Needs (SEN) are defined broadly as having: **a)** “a restriction in the capacity of the person to participate in and benefit from education on account of an enduring physical, sensory, mental health or learning disability, or any other conditions which results in a person learning differently from a person without that condition.”	No empirical evidence of students in the Republic of Ireland with a history of cancer being granted access to special education and related services.	20 March 2018

**COUNTRY**	SURVIVAL RATE*	**SPECIAL EDUCATION/DISABILITY RIGHTS LEGISLATION**	**POTENTIAL APPLICATION TO CHILDREN TREATED FOR CANCER**	**EFFECTIVENESS**	RATIFICATION OF THE UN CRPD**

United Kingdom: England	85%	Special Education Needs and Disability Regulations (2014)	Children treated for cancer fall under protected disability status. Students may qualify for special education as a child or young adult of compulsory school age with “a significantly greater difficulty in learning than the majority of others of the same age.”	No empirical evidence of students in the United Kingdom with a history of cancer being granted access to special education and related services.	8 June 2009
	
United Kingdom: Scotland		Additional Support for Learning (Scotland) Act (2004)	a prescribed pre-school child, a child of school age or a young person receiving school education, provision which is additional to, or otherwise different from, the educational provision made generally for children or, as the case may be, young persons of the same age in schools (other than special schools)		
	
United Kingdom: Wales		Special Education Needs and Disability Regulations (2014), Additional Needs Supports for Learning (Wales) Act (2018)	child of compulsory school going age or person over that age as having a “significantly greater difficulty in learning that the majority of others of the same age, or has a disability for the purposes of the Equality Act 2010 which prevents or hinders him or her from making use of facilities for education or training of a kind generally provided for others of the same age in mainstream maintained schools or mainstream institutions in the further education sector		
	
United Kingdom: Northern Ireland		Special Needs and Disability (Northern Ireland) Order (2005), Special Needs and Disability Act (2016)	a child has special educational needs if they have a learning difficulty, meaning, “a significantly greater difficulty in learning than the majority of children his age, he has a disability which either prevents or hinders him from making use of educational facilities of a kind generally provided for children of his age in ordinary schools		

**COUNTRY**	**SURVIVAL RATE**	**ISS/DISABILITY RIGHTS LEGISLATION**	**POTENTIAL APPLICATION TO CHILDREN TREATED FOR CANCER**	**EFFECTIVENESS**	RATIFICATION OF THE UN CRPD**

United States	85%	Individuals with Disabilities Education Act (2004), Americans with Disabilities Act (1990).	Potential qualifying special education disability category of “Other Health Impairment”: “having limited strength, vitality, or alertness, including a heightened alertness to environmental stimuli, that results in limited alertness with respect to the educational environment that – (i) Is due to chronic or acute health problems such as asthma, attention deficit disorder, or attention deficit hyperactivity disorder, diabetes, epilepsy, a heart condition, hemophilia, lead poisoning, leukemia, nephritis, rheumatic fever, sickle cell anemia, and Tourette syndrome; and (ii) Adversely affects a child’s educational performance.”	Limited evidence that students in the United States with a history of cancer are being granted access to IEPs/ILPs.	Signed 30 July 2009, but never ratified


*Childhood cancer survival rate based on 5 year post-diagnosis data. **United Nations Convention on the Rights of Persons with Disabilities

## Research Strategy

### Inclusion Criteria

Inclusion criteria included three parts; 1) countries must have a five-year childhood cancer survival rate of at least 80%, 2) countries must have a national population of children under the age of 15 of at least 700,000, 3) countries must have national and state/providence/territory laws specific to educational protections for children with disabilities written in English. Our inclusion criteria required that countries have a childhood cancer five-year survival rate of at least 80%. Childhood cancer survival rates of this magnitude are a recent development within the last 20–30 years. However, these national survival rates are not universal and rates of 30% are unfortunately not uncommon. We elected to focus on countries with high rates of survivorship as these are the countries well-suited to focusing attention beyond the treatment of cancer toward quality of life during and after treatment, including education. Countries with legislation written in English was used as an inclusion criterion to address restraints of the current research team.

### Determining Survival Rates

The Social Demographic Index (SDI) generated by the Global Burden of Disease (2019) study was used as a mechanism for determining which countries had a 5-year survival rate of 80% or higher. The SDI is a composite of indicators of a country’s level of development that is strongly correlated with health outcomes, including childhood cancer survival rates. According to the World Health Organization (WHO), countries who are ranked in the “high SDI” quintile have average five-year survival rates of 80%. The Global Burden of Disease listed 35 independent nations within the high SDI quintile. Childhood cancer survival rates were then corroborated through published health statistics of each country.

### Determining Language of Written Law that impacts ISS

The 35 countries identified by the GDB (2019) as having “High SDI” were next sorted by the official language of their national legislation. Seven countries met this inclusion criteria; Australia, Bermuda, Canada, New Zealand, Republic of Ireland, the United Kingdom (which is comprised of England, Scotland, Wales, and Northern Ireland, but reports their health data collectively as one nation), and the United States. Some countries, such as the Republic of Ireland officially publish their laws in more than one language, but as official English legislation was to meet the limitations of the research team, countries multiple official languages that includes English were readily included. At this point New Zealand was excluded because they have no current national laws that create in-school supports for children with disabilities. Rather, New Zealand has moved to legislation that states all students have a right to full inclusion, but does not offer clear pathways for guaranteeing ISS (Community Law 2021; Kearney, 2016).

### Determining Population Demographics

The final inclusion criterion was to have a population of children 15 years of age and younger that reached at minimum 700,000. This figure was determined by looking at childhood cancer rates. A population of 700,000 means approximately 100 childhood cancer cases per-year, creating large enough group of children to warrant consideration of formal educational policy. At this point Bermuda was excluded due to its small population of children 15 years of age or younger. Notably, the WHO and GBD (2019; 2021) both report demographics and statistics for the United Kingdom (UK) as a whole rather than for each of the four countries comprising the UK (England, Northern Ireland, Scotland, and Wales). The UK also reports all childhood cancer statistics as a whole rather than providing breakdowns per-country. Therefore, while three of the four countries comprising the UK would not have populations of 15 years of age or younger large enough for inclusion in this study, we have decided to follow the reporting of the WHO and GDB and report on the UK as a unit.

### Document Search Strategy

Of the countries identified, screening and selection were conducted based on the alignment of retrieved documents with the research question and inclusion/exclusion criteria, involving the review of titles, abstracts, and full texts. Official government websites, legislative bodies’ websites, and organizations related to education, health, and disability rights in the selected countries were explored for relevant legislation, policy documents, and guidance. Also, databases such as PubMed, PsycINFO, ERIC, and Google Scholar were chosen in order to select relevant documents and research.

A comprehensive list of search terms related to childhood cancer survivorship, special education rights and provisions, as well as quality legislation was developed, including variations and combinations of keywords such as “childhood cancer survivorship,” “special education,” “disability rights”, “special education law”, “special education policy”, “students with disabilities”, “students with special education needs” and “special education services”. The search strategy used Boolean operators (AND, OR, NOT) to combine search terms effectively. Inclusion criteria were defined, encompassing documents published in English, legislation or policy guidance related to special education and equality legislation, and documents specifically focusing on childhood cancer survivorship within the selected countries. These steps collectively constituted a thorough search strategy to find relevant documents on legislation and policy related to childhood cancer survivorship and special education rights and provisions in the selected countries.

## Legal Summaries

Any comparison of legislation across countries requires some note about systems of government. Three of the countries examined here (Australia, Canada, and the United States) are organized in a federal system, with power distributed between the national level (laws applicable to the whole of the country), the state/providence/territory level, and the local level (laws applicable to smaller jurisdictions within states/providences/territories). For each of these three countries we sought to examine national laws (when available) with respect that state/providence/territory laws may provide additional protections to students.

Ireland is a unitary state and full legislative powers lie with the central government. Therefore, in Ireland, national laws apply to the whole of Ireland with no deviation between local authorities. Because the UK is a constitutional monarchy and parliamentary democracy it is considered an asymmetrically decentralized unitary state, comprised of England and three countries with devolved governments: Scotland, Wales and Northern Ireland. Each devolved country has its own national identity and distinct governing systems. Hence, for the UK, we sought to examine the national law of England and that law as acted upon, or not, in the devolved countries of Scotland, Wales and Northern Ireland.

### Country: Australia

#### ISS Law Summary

Education of students with a disability in Australia is regulated by the Disability Standards for Education (DSE) (Government of Australia 2014) which is subordinate to the Disability Discrimination Act (DDA) (Government of Australia 1992; NCCD, 2022). The DDA requires that schools make “reasonable adjustments” for students with disabilities of which the legislation lists nine categories of eligibility (Dickson, 2022). The DSE sets criteria for the necessity of an Individualized Education Plan (IEP) or Individualized Learning Plan (ILP). While the DDA includes nine categories of recognized disability, the Nationally Consistent Collection of Data on School Students with Disabilities (NCCD), an Australian government initiative, classifies these nine categories of disability included in the DSE into four classifications; physical disabilities, cognitive disabilities, sensory disabilities, and social/emotional disabilities (NCCP, 2022a). As previously mentioned, Australia is a commonwealth, a federal style of government with power distributed between the national government and these are national laws which are enacted at the state level which may have additional protections for students.

Of these eligibility criteria, students with cancer are most likely to qualify as a student with a disability that can be described as “the presence in the body of organisms causing disease or illness” (Government of Australia 1992), as this category best describes a student with an illness. The NCCP (2022a) places within its condensed category of ‘physical’ disability. However, for students who have completed cancer treatment and are in remission, this category does not accurately describe the cognitive late effects of cancer and treatment that may call for ISS. Thus, for students who have transitioned into remission, the DDS category of “a disorder or malfunction that results in the person learning differently from a person without the disorder or malfunction” may be a better fit. The NCCD (2022) places within its condensed category of ‘cognitive’ disability.

#### Feasible ISS Protections for Children Treated for Cancer

The DDA clearly offers disability rights protections from school-based discrimination for children with a “disease or illness” (Dickson, 2022) which fits well for children being treated for cancer. However, as impacts of neurocognitive late effects of cancer differs from the impact of children who are ill and in treatment, survivors may be better covered under the category described as “a disorder or malfunction that results in the person learning differently from a person without the disorder or malfunction.”

#### Evidence of Effectiveness of ISS for Children Treated for Cancer

There is limited published evidence that Australian students with a history of cancer are being given access to IEPs/ILPs. In a study of families representing 80 children treated for cancer, reflecting national cancer statistic, by Donnan, et al. (2015) only 6.7% of parents reported that their child was found eligible for ILPs despite 48% of parents reporting significant difficulties with cognition and academic skills. Qualitative studies of post-cancer schooling experiences provide conflicting information; McLoone, et al. (2013) discusses that schools are supportive toward students during reentry to school post-treatment, but makes no mention of ISS, even with regard to students who are noted as having neurocognitive late effects of cancer and are struggling academically. Cheung, et al. (2014) paints a different picture with the needs of students with a neurocognitive late effect of cancer and treatment being given adequate support through IEPs which were well matched to the neuropsychology reports.

### Country: Canada

#### ISS Law Summary

The authority to govern education in Canada is held at the provincial/territory, rather than the national level. Canada consists of ten provinces and territories and therefore has 10 separate laws pertaining to the education of students with disabilities, yet each province and territory is beholden to the Canadian Charter of Rights and Freedoms (Government of Canada 1981; Sokal & Katz, 2020) in which section 15 states; “Every individual is equal before and under the law and has the right to the equal protection and equal benefit of the law without discrimination.” Disability is specifically listed within the Canadian Charter of Rights and Freedoms as a protected identity.

Thus, equal protection under the law, including those with disabilities, guides the education law and policies developed by each Canadian provinces and territory (CEC, 2023) creating an expectation that all school districts ensure equity in public education for students with disabilities. Given that special education laws vary significantly by Canadian province/territory, we will look at Canada’s most populated province, Ontario. Ontario’s Education Act (Government of Ontario 1990) dictates that special education is to be provided to students identified as “exceptional pupil[s]” which is defined by “behavioral, communicational, intellectual, physical or multiple exceptionalities [that] are such that he or she is considered to need placement in a special education program by a committee” (Government of Ontario 1990; LDAO, 2003).

Of these eligibility criteria, students who have been treated for cancer are most likely to qualify under the category of *communication* and subcategory of *learning disability* which is described as “a neurodevelopmental disorder that persistently and significantly has an impact on the ability to learn and use academic and other skills” (Government of Canada 2023). This subcategory further describes this category to include “phonological processing; memory and attention; processing speed; perceptual-motor processing; visual-spatial processing; executive functions” which are the most common types of neurocognitive late-effects of childhood cancer and treatment (Jacobson et al., 2020).

#### Feasible Special Education Protections for Children Treated for Cancer

Although the description of learning disability within Ontario’s education law includes a list of the cognitive skills most impacted by neurocognitive late-effects of childhood cancer and treatment, it is unclear if school teams would agree to label neurocognitive late-effects as neurodevelopmental disorder, which is a key aspect of the subcategory’s definition. Of Canada’s 10 provinces/territories, Ontario’s special education legislation is an example of the most robust, therefore families may face increased difficulties in having children with a history of cancer and treatment found eligible for special education programing in other areas of Canada (LDAO, 2003; Lum et al., 2019).

#### Evidence of Effectiveness of Special Education Access for Children Treated for Cancer

Families report difficulty accessing proper educational support for students with a history of cancer and treatment (Bruce et al., 2012). Currently, while the Canadian Cancer Society (2024) provides information about learning difficulties related to neurocognitive late effects of childhood cancer and treatment, there is no mention of pursuing special education as a recommended support.

### Country: Republic of Ireland

#### ISS Law Summary

Special Education in Ireland is regulated by the Education for Persons with Special Educational Needs (EPSEN) Act 2004 (Government of Ireland 2004). This legislation sets out the provision of appropriate education in an inclusive environment requiring that “people with special educational needs have the same right to an education as do their peers who do not have such needs” (Government of Ireland, 2004, p. 5). A critical component of EPSEN is IEPs or Student Support Files (SSF), their primary purpose being to facilitate the planning for, review and monitoring of a student’s progress. Student goals within the IEP are to be achieved over a period not exceeding 12 months and each IEP must be reviewed at least once a year. Unfortunately, the sections of EPSEN to confer statutory entitlements to educational assessments for all children with Special Educational Needs (SEN), a statutory IEP, are still not ratified (NCCP, 2022a). It is also important to note that a review of EPSEN commenced in 2022 to ensure the adequacy of the Act in the context of Irish society today, and to provide assurance that the law governs the provision of education for children with SEN (2004).

EPSEN, in its current iteration, defines special education needs as a restriction in the capacity of the person to participate in and benefit from education on account of an enduring physical, sensory, mental health or learning disability, or any other condition which results in a person learning differently from a person without that condition and cognate words shall be construed accordingly (p. 6).

#### Feasible Special Education Protections for Children Treated with Cancer

Apart from the government’s broad definition of SEN, EPSEN does not include separate eligibility categories for students to receive support and services. However, the language of the current definition “or any other condition which results in a person learning differently from a person without that condition” suggests that EPSEN and therefore special education protections should apply to children who face barriers to their learning and poor educational outcomes due to their history with cancer and treatment. These children are further protected by Ireland’s Equal Status Acts (2000–2018). The Acts places certain obligations on schools in relation to how they deliver their services to ensure they do not discriminate against the nine grounds of discrimination as set out in the Acts. Disability is one of these grounds and is defined in part as “the presence in the body of organisms causing, or likely to cause, chronic disease or illness” (Government of Ireland 2000, p. 5). A consideration of EPSEN in conjunction with this definition would give legislative rights to children treated with cancer to special education support. Furthermore, the current Special Education Teachers Resource allocation gives schools the autonomy to use their given allocation in a manner that suits the needs of their students best, giving schools agency to direct of their resources to support children treated with cancer. However, the wording “presence in the body” suggests a sense of current illness, rather than survivorship. This could lead to schools making resource decisions based on the status of the child’s medical condition rather than their learning needs post treatment. The current definition’s language does not account for the lasting negative impact of cancer treatment on the child in remission or survivorship that continues to cause barriers to learning and poor educational outcomes for cancer survivors.

#### Evides this used a gnce of Effectiveness of Special Education Access for Children Treated for Cancer

The National Cancer Control Programme (2021, 2022a, 2022b) states that a cancer diagnosis and subsequent treatment challenge or delay Adolescents and Young Adults capacity to achieve developmental milestones such as completing education. The Health Service Executive (2023) notes that challenges, such as any late effects of treatment, can surface over time. The literature (HSE, 2023; Li et al., 2023; Marchak et al., 2022; Shi et al., 2022) identifies late effects contributing to limited access to education. However, there appears to be no literature in Ireland on access to special education resources and services for children with cancer or cancer survivors.

### Country: United Kingdom: England

#### Special education Summary Law

Special education in England is regulated by The Special Educational Needs and Disability (SEND) regulations (UK Government 2014b), which sets out the main regulations underpinning The Child and Families (CARE) Act (UK Government 2014a), and Special Educational Needs and Disability (SEND) Act (UK Government 2001). Statutory documents arising from these are ‘Special educational needs and disability code of practice: 0 to 25 years’ (Department of Education 2015) and ‘Supporting Pupils at School with Medical Conditions’ (Department of Health 2015). This legislation sets out a clearer focus on having higher expectations and on improving educational outcomes for children and young adults with SEND, and the participation of these children and young people, and their parents/guardians, in decision making about their education.

In terms of provision for children and young adults with SEND, both the Act and recommendations outline the process for an Education and Health Care (EHC) needs assessment, securing the provision recommended because of these assessments and agreeing the required budgets for such provision. A request for an EHC plan must prove that the student’s needs surpass what the school can provide. EHC plans are legal documents for which the Local Authorities in England are responsible. While IEPs are not mentioned in this legislation, or the accompanying SEND Code of Practice (Department for Education 2015), schools can create and put IEPs in place to support any child and are a precursor to evidencing the need for an EHC plan or where a student does not meet the criteria for an EHC plan.

SEND, as defined in this legislation, is a child or young adult of compulsory school age with a learning difficulty or disability whereby they have “(a) a significantly greater difficulty in learning than the majority of others of the same age, or (b) has a disability which prevents or hinders him or her from making use of facilities of a kind generally provided for others of the same age in mainstream schools or mainstream post-16 institutions” (UK Government 2014a).

#### Feasible Special Education Protections for Children Treated with Cancer

In addition to CAFA’s broad definition of SEND, it also makes provision for children with medical conditions. In England, a person is automatically protected under the disability definition within the Equality Act (UK Government 2010) from the day they are diagnosed with cancer. Statuary guidance ‘Supporting pupils at school with medical conditions’ (Department of Health 2015) sets out clear expectations for schools and local authorities. It recognizes that a child’s health may deteriorate while they attend school, that students with ongoing and complex health conditions may need continuing support, their medical and care needs may change unpredictably over time, and that frequent, short-term and extended absences are often a result of complex health conditions. Furthermore, it recognizes that there are social and emotional implications associated with medical conditions. Finally, it recognizes that these factors accumulatively affect a child’s engagement in learning and their educational outcomes. To effectively manage this the guidance points out that students with medical conditions who are attending school “should be properly supported so that they have full access to education, including school trips and physical education” (Department of Health 2015, p. 4) and that governing bodies must ensure that these supports are in place and that school leaders are liaising with relevant health and social care professionals to so as to understand the medical conditions to effectively support the student.

### Country: United Kingdom: Scotland

#### Special Education Summary Law

In Scotland, special education law is primarily governed by the Education (Additional s this guidance code of practice. The legislation sets out the provision for identifying and addressing the Additional Support Needs of children and young people who face a barrier, or barriers to learning. The Act is structured around the concept of support being needed for any reason, for short or long-term periods in accordance with the individual learning needs of the child or young person. IEPs are non-statutory documents in Scotland that, as in other countries, outline the provision plan for individual students with ASN to achieve their specified learning outcomes. For students with more complex or multi-factor needs where interagency support is needed a Coordinated Support Plan is developed. This statutory document is similar to an IEP but with the involvement of agencies outside of the school.

The Additional Support for Learning (Scotland) Act (Government of Scotland 2017a) defines Additional Support Needs as

in relation to a prescribed pre-school child, a child of school age or a young person receiving school education, provision which is additional to, or otherwise different from, the educational provision made generally for children or, as the case may be, young persons of the same age in schools (other than special schools) under the management of the education authority for the area to which the child or young person belongs (p. 1)

#### Feasible Special Education Protections for Children Treated with Cancer

As in England, a Scottish person is automatically protected under the definition of disability within the Equality Act (2010) (UK Government 2010) from the day they are diagnosed with cancer. Additionally, the Education (Scotland) Act 2016 (Government of Scotland 2016) states that children and young adults experiencing health and wellbeing challenges continue to have the same rights to education as all children. ‘Supporting children and young people with healthcare needs in schools’ (Government of Scotland 2017b) provides guidance to national health services, education authorities and schools. Like England and Wales, this guidance covers the rights of children and young people with health conditions, developing policies and procedures, and roles and responsibilities of schools. While this guidance refers to legislation it is not a statutory document.

### Country: United Kingdom: Wales

#### Special Education Summary Law

As in England, special education in Wales is regulated by The Special Educational Needs and Disability (SEND) Regulations 2014, which sets out the main regulations underpinning the Child and Families Act 2014 (CAFA), and the Additional Learning Needs and Education Tribunal (Wales) Act 2018 and its subsequent The Additional Needs (Wales) Regulations (Government of Wales 2021a) and The Additional Learning Needs (ALN) Code for Wales (Government of Wales 2021b). This statutory framework sets out a unified legislative approach to supporting all children of compulsory school age or below with ALN, and in supporting young people with ALN who are in school, a Pupil Referral Unit or further education. Under this framework IEPs are replaced with an Individual Development Plan (IDP). IDPs are legal documents which describe the child’s ALN, set out what is required to support their needs with details of targets, strategies and progress review. The ALN Code defines a learning difficulty or disability for child of compulsory school going age or person over that age as having a

significantly greater difficulty in learning that the majority of others of the same age, or has a disability for the purposes of the Equality Act 2010 which prevents or hinders him or her from making use of facilities for education or training of a kind generally provided for others of the same age in mainstream maintained schools or mainstream institutions in the further education sector ( p. 28).

#### Feasible Special Education Protections for Children Treated with Cancer

As in England, a Welsh person is automatically protected under the disability definition within the Equality Act (2010) from the day they are diagnosed with cancer. ‘Supporting Learners with Healthcare Needs’ (Government of Wales 2017) provides both statutory guidance and non-statutory advice to assist Local Authorities, education settings, governing bodies, and other relevant education and health professionals and organizations. This guidance is similar to England’s ‘Supporting Pupils at School with Medical Conditions’ (Department of Health 2015), setting out the same clear expectations for schools and local authorities. It goes further in its guidance on training for staff and in the role of the learner in managing their own healthcare needs. It notes the need for the consideration of flexible curriculum delivery and a collaborative approach in decision making.

### Country: United Kingdom: Northern Ireland

#### Special Education Summary Law

Special Education in Northern Ireland is governed by The Special Needs and Disability (Northern Ireland) Order (SENDO) (Government of Northern Ireland 2005) and The Special Educational Needs and Disability Act (Northern Ireland) (Government of Northern Ireland 2016). This legislation sets out the roles and responsibilities of schools and Education Authorities (EA) in the identification, assessment and provision for children with SEN so to ensure children with SEN are not substantially disadvantaged in their education. A key provision in this legislation is the statutory assessment and statement of special educational needs where the support offered in school is insufficient to support the student’s needs. The statement of educational needs gives general information about the child and the advice received by the Education Authorities following the statutory assessment. This includes a description of the child’s educational and non-educational needs, provision to be given to support the child’s needs, the type and name of school recommended or preferred for the child and out of school provision. This process, in addition to other guidance is outlined in The Code of Practice on the Identification and Assessment of Special Educational Needs (1998) which falls under The Education (Northern Ireland) Act 1996 (Northern Ireland Orders in Council 1996).

Special Educational Needs and disability are defined in separate Acts in Northern Ireland. The Education (Northern Ireland) Order 1996, a child has special educational needs if they have a learning difficulty, meaning:

a significantly greater difficulty in learning than the majority of children his age, he has a disability which either prevents or hinders him from making use of educational facilities of a kind generally provided for children of his age in ordinary schools, or he has not attained the lower limit of compulsory school age and is, or would be if special educational provision were not made for him, likely to fall within sub-paragraph (a) or (b) when he is of compulsory school age (p. 2).

The Disability Discrimination Act 1995 defines disability as having “a physical or mental impairment which has a substantial and long-term adverse effect on his ability to carry out normal day-to-day activities” (Northern Ireland Orders in Council 1996, p. 2), a definition which broadly aligns with the rest of the United Kingdom (England, Wales and Scotland).

#### Feasible Special Education Protections for Children Treated with Cancer

While Northern Ireland has broadly the same definition of disability as the Equality Act 2010, equality law has devolved in Northern Ireland since 1998. This means that the protections people with cancer receive under the Equality Act 2010 in England, Scotland and Wales are not extended to Northern Irish citizens. However, the language of the current definitions of SEN and disability, “a significantly greater difficulty in learning than the majority of children his age” and “a physical or mental impairment which has a substantial and long-term adverse effect on his ability to carry out normal day-to-day activities” (Government of Northern Ireland 1998, p. 1) suggests that special education should apply to children where their cancer and effects of treatment significantly impact their ability to carry out normal day-to-day activities such as going to school. Furthermore, the ‘The Code of Practice on the Identification and Assessment of Special Educational Needs’ (1998) directs schools who suspect a child’s learning difficulty may be due to a medical condition to seek consent to consult with the child’s General Practitioner. The Code notes that the impact of a medical condition or illness secondary effects can impact learning and lead to difficulties. However, while The Code acknowledges that leukemia and other childhood cancers, and their treatment, may significantly impact the child’s participation in the curriculum and school life, the language “will periodically affect their ability to participate fully” (Government of Northern Ireland 1998, p. 11) could be interpreted as an intermittent rather than long-term negative affect of cancer and cancer treatment on a child’s participation in learning that exists after the child is considered in remission or cancer free.

#### Evidence of Effectiveness of Special Education Access for Children Treated for Cancer

While England, Wales and Scotland have legislated for the provision of special education access for children treated with cancer, a search of the literature did not yield any evidence of the effectiveness of this statutory access. However, government statutory and non-statutory guidelines and templates to support children with medical conditions including the responsibility of school leaders to understand the need of the child with, in this instance, cancer and to support them accordingly suggests that access and support is occurring at some level. Gaps between the legislation and guidance for children with healthcare in school, and actual practice have been identified in Scotland (CHS, 2023). Children’s Health Scotland highlights that the impact of health on the child’s education is often underestimated, and miscommunication can result in a child not receiving educational provision that equates to their statutory rights. They also note the length and complexity of legislation and guidance can lead to families, education authorities and school staff having insufficient awareness of the educational rights of students with health conditions and the responsibilities of authorities and schools to deliver appropriate education and care to these children. Furthermore, while The Code of Practice in Northern Ireland refers to medical conditions and cancer, limited information on what this looks like in practice was found. More research into what is happening and to what level, especially for children treated with cancer, is needed.

### Country: United States

#### Special Education Law Summary

Special Education in the United States is regulated by the Individuals with Disabilities Education Act (IDEA) (2004), a national law re-established in 2004. This legislation protects the rights of primary and secondary education students to a “free and appropriate public education” in the “least restrictive environment” possible while supporting their educational progress. Under IDEA, students are provided with an Individualized Education Program (IEP) which includes annual goals, accommodations, modifications, and provisions for access to related services such physical therapy, occupational therapy, speech and language services, and counseling.

The IDEA includes thirteen separate eligibility categories through which students can be found eligible for special education and related services. Each category includes its own inclusion and exclusion criteria. IEP teams are asked to consider which category a student with a suspected disability may qualify for special education services under and then use that category’s criteria to determine a student’s special education eligibility (Dombrowski, 2020). Of these eligibility criteria, students treated for cancer are most likely to qualify under the IDEA category of Other Health Impairment (OHI). This category lays out the eligibility criteria as:

“Other health impairment means having limited strength, vitality, or alertness, including a heightened alertness to environmental stimuli, that results in limited alertness with respect to the educational environment, that:

(i) Is due to chronic or acute health problems such as asthma, attention deficit disorder or attention deficit hyperactivity disorder, diabetes, epilepsy, a heart condition, hemophilia, lead poisoning, leukemia, nephritis, rheumatic fever, sickle cell anemia, and Tourette syndrome; and
(ii) Adversely affects a child’s educational performance.” (United States Congress 2004, p. § 300.308)

“Limited strength, vitality, or alertness” are not further defined within the IDEA but have been interpreted as difficulty completing routine physical tasks within the school setting, fatiguing physically and or mentally more easily than non-disabled peers, and difficulty attending to relevant stimuli, sustaining attention, or attending to the same amount of information as non-disabled peers (Carey et al., 2023).

#### Feasible ISS Protections for Children Treated for Cancer

The IDEA’s eligibility criteria of OHI specifically cites leukemia as an example health condition. This suggests that special education protections should be extended to students who show poor educational performance related to their history of cancer and treatment. Leukemia stands out, however, as the only health condition listed as an example within the legislation that is often cured or put into remission (Carey et al., 2023). This means that school teams may discount the eligibility of a student in remission or post-treatment, even when the negative effects of cancer and treatment are still causes learning difficulties. Even without the example of leukemia within the legislation, children who have been treated for cancer frequently meet the criteria of having “limited strength, vitality, and alertness” as the most common neurocognitive late effects of childhood cancer and treatment include fatigue, decreased processing speed, and decreased attentional control (Ruble et al., 2023).

#### Evidence of Effectiveness of ISS Access for Children Treated for Cancer

There is limited evidence that students treated for cancer are being found eligible for special education to the degree that would be anticipated – even with the IDEA’s inclusion of leukemia as an example of a qualifying condition under the category of OHI (Jacobson et al., 2020). The literature suggests that there are multiple potential factors contributing to limited access to special education for children treated for cancer. These include, limited understanding of the impact of cancer and treatment on the developing brain among educators (Brand et al., 2017), limited communication about the neurocognitive late effects of cancer on the part of oncology providers (Thornton et al., 2022), and the obtuse phrasing of the OHI eligibility criteria within the IDEA (Dombrowski, 2020).

## Discussion and Conclusion

There is a reasonable expectation for children treated for cancer to be found eligible for special education services in the countries reviewed. These countries, however, were variable in how explicitly legislation and guidelines address children treated for cancer. For example, legislation in the United States explicitly gives an example of leukemia as a qualifying health condition for special education services while the Republic of Ireland’s legislation does not mention cancer, but still describes conditions that would encompass neurocognitive late effects of cancer and treatment. While the same legislation existed for England, Wales, and Scotland, each country’s guidelines for children with medical conditions differed. Some legislation had vague or mischaracterized information about cancer, for example, in the United States, while leukemia is mentioned, it is unclear if this pertains to children after treatment has concluded, while in Northern Ireland the language of their code of practice could be interpreted as the effects of cancer being intermittent rather than life long.

Even with legislation in place in each of the reviewed countries, there was very limited evidence of children treated for cancer accessing special education services. For example, in the United States, which had the most explicit legislation supporting the access to ISS for children treated for cancer, the literature pointed to extreme difficulties in accessing special education services. Literature from Canada and Australia note the same difficulties in accessing ISS after cancer treatment. Despite organizations such as NCCP and the HSC in Ireland noting the challenges in education for children treated for cancer, the literature on access to special education for these children in Ireland appears non-existent.

Considering the findings of this review it appears that while eligibility under the law is at times unclear, the larger barrier to accessing ISS after cancer treatment is the siloed nature of education and pediatric medicine in the pursuit of supporting these children (Martinez-Santos et al., 2021; Pini et al., 2016) as well as a lack of understanding of the long-term effects of cancer and cancer treatment on children’s educational experience. As noted, Children’s Health Scotland’s (2023) report indicates extreme gaps between legislation guidance, understanding, knowledge, and practice in addition to stressing the negative impact of misinformation and miscommunication in accessing special education services. If the existence of legislation and guidelines to allow for children treated for cancer to access ISS is not the primary barrier to equitable education, then the fields of special education and pediatric medicine must determine how best to address the identified gap and reconcile the educational needs of childhood cancer survivors (Plage et al., 2022). ISS legislation should recognize the unique needs of these individuals with the requisite measures in place to do so, ensuring access to meaningful education and supportive services.

While the ISS legislation and guidelines for each country included nebulous language, the equality acts of each country enshrine the rights of people with disabilities (i.e., medical conditions, such as cancer) to public services such as education. Therefore, it is incumbent upon the education and medical communities of these countries to develop systems of collaboration and professional learning across teams to reduce the unintended harm done by exclusionary discrimination in the educational setting.
